# Prevalence, Magnitude and Methods of Rapid Weight Loss Reported by Male Mixed Martial Arts Athletes in Ireland

**DOI:** 10.3390/sports7090206

**Published:** 2019-09-09

**Authors:** John Connor, Brendan Egan

**Affiliations:** 1 School of Health and Human Performance, Dublin City University, Dublin D09 V209, Ireland; 2 National Institute for Cellular Biotechnology, Dublin City University, Dublin D09 V209, Ireland

**Keywords:** combat sports, dehydration, hot bath, weight category, weight-making, water loading

## Abstract

Rapid weight loss (RWL) is frequently practiced in weight category sports, including Mixed Martial Arts (MMA). The aim of the present study was to describe self-reported methods of RWL in a sample of competitive MMA athletes comprising of both amateur and professional fighters. The previously-validated Rapid Weight Loss Questionnaire, with the addition of questions on water loading and hot salt baths, was completed anonymously online by athletes (n = 30; all male, n = 15/15 professional/amateur) from MMA clubs around Dublin, Ireland. All but one (97%) of the athletes surveyed lost weight in order to compete, with the average weight loss being 7.9% ± 3.1% of habitual body mass. The RWL score (mean ± SD) for this sample was 37.9 ± 9.6, and a tendency for higher [6.0 (95%CI; −1.1, 13.1) (*p* = 0.093; *d* = 0.64)] RWL scores for professional (40.8 ± 8.9) compared to amateur (34.8 ± 9.6) athletes was observed. Frequencies of “always” or “sometimes” were reported as 90% for water loading, 76% for hot salt baths and 55% for 24 h of fasting. Fellow fighters (41%) and coaches/mentors (38%) were “very influential” on RWL practices of these athletes, with doctors (67%), dietitians (41%), and physical trainers (37%) said to be “not influential”. RWL is highly prevalent in MMA across both amateur and professional athletes, and RWL scores are higher than other combat sports. Water loading and hot salt baths are amongst the most commonly used methods of RWL despite little research on these methods for body mass reduction or effects on performance in weight category sports.

## 1. Introduction

Rapid weight loss (RWL) is frequently practiced in sports that have weight class restrictions [1,2,3,4,5]. Many of these sports include combat sports such as wrestling, judo, boxing and taekwondo, as well as other mainstream sports such as horse riding and rowing [2,6]. RWL generally refers to the methods employed by an athlete in reducing body mass in the final one to two weeks before competition, and typically averages ~2% to 10%, depending on the sport [1,2,3,4,5]. Subsequent to the weigh-in, combat sport athletes generally proceed to regain often the majority of this weight from within a few hours up to 36 h before competing [7,8,9]. RWL followed by rapid weight regain is employed, especially in combat sports, as a means of gaining a size and/or strength advantage over an opponent as the heavier fighter is generally seen to have an advantage [1,3,8,10].

Mixed martial arts (MMA) is a combat sport comprised of styles of various martial arts and involves striking, grappling, wrestling and submission techniques [11]. MMA athletes are required to compete under specific weight categories, namely: atomweight, 105 lbs (47.6 kg); strawweight, 115 lbs (52.2 kg); flyweight, 125 lbs (56.7 kg); bantamweight, 135 lbs (61.2 kg); featherweight, 145 lbs (65.8 kg); lightweight, 155 lbs (70.3 kg); welterweight, 170 lbs (77.1 kg); middleweight, 185 lbs (83.9 kg); light-heavyweight, 205 lbs (93.0 kg); heavyweight, 205–265 lbs (93.0–120.2 kg); and super-heavyweight, no limit. In professional bouts for MMA, the timeline between weigh-ins and fight time can vary depending on the organisation sanctioning the fight. All professional organisations have weigh-ins on the day before the fight. For the majority of organisations, weigh-ins are at least 24 h before the fight and up to 36 h beforehand. The timeframe for amateur MMA fights again depends on the organisation sanctioning the bout. Many organisations will follow the same outline as the professional bouts on their card (24 to 36 h before the fight), but under new rules set forth by the International Mixed Martial Arts Federation (IMMAF), weigh-ins for amateur fights are on the morning of competition.

MMA was established on the international stage as the Ultimate Fighting Championship (UFC) in 1993, but despite being one of the fastest-growing international sports [12], only recently have reports begun to emerge on the weight-making practices of these athletes [7,8,13,14,15,16,17]. One survey described MMA athletes losing 9% ± 2% of body mass in the week before a fight, and a further 5% ± 2% in the 24 h before weigh-in [16]. This is achieved due to employing one or all of the following methods: water loading, fluid restriction, prescription and over-the-counter diuretics, complete fasting or low carbohydrate diets in the final 3 to 5 days prior to weigh-in [16]. Such drastic methods for RWL result in 100% of athletes being dehydrated to various degrees at the official weigh-in [7,13], and 14% [7] and 39% [13] remaining dehydrated when measured in the final 2 h pre-fight.

Considering the increasing popularity of MMA, but documented adverse health outcomes and deaths attributed to RWL practices [16,17,18], the creation of bodies such as Safe MMA recognised that RWL practices may increase the risk of injury and health consequences. Indeed, there have been calls to ban RWL in combat sports, partly because of the potential health risk to the athlete [19]. Conversely, the case has been made that a well-designed RWL strategy supported by appropriate recovery and weight regain strategies—when the time from weigh-in to competition allows—may confer a performance advantage [5]. While the data across weight category sports as a whole remain equivocal [3,5], weight regain has been linked to a performance advantage in judo [10] and MMA [8]. Further studies are needed to characterize the prevalence and methods of RWL in MMA, with additional work then required to establish the safety, or otherwise, of these methods. Therefore, the aim of the present study was to describe self-reported methods of RWL in a sample of competitive MMA athletes comprising of both amateur and professional fighters based in Dublin, Ireland. 

## 2. Materials and Methods

### 2.1. Study Design and Participants

The study was approved by the Research Ethics Committee at the Dublin City University (DCU), Ireland (permit: DCUREC 2017_055) in accordance with the Declaration of Helsinki. Participants, all of whom were male, were recruited from several MMA clubs around Dublin that are associated with Straight Blast Gym (SBG), the largest MMA gym franchise in Ireland. Participants were invited to participate in a survey of current and previous weight-making practices via the fighters’ private page on Facebook. Participants clicked via a link that gave them access to the anonymous online questionnaire. A participant information leaflet was presented on arrival to the page, after which participants needed to provide consent via a tick box option in order to proceed to the questionnaire. Prior to commencing the questionnaire, RWL was defined to the participants as reducing body mass by 5% to 10% in seven days or less.

The private Facebook page is for active fighters only (i.e., have previously competed and are continuing to prepare for future fights), and has a membership of fifty athletes with an even distribution of amateur and professional fighters. Thirty athletes (60%) completed the online survey, with a final split of n = 15 amateur fighters, and n = 15 professional fighters. Professional and amateur status was self-reported and categorised based on the rules set under which they fought at the time of the questionnaire being administered. The major distinctions between the respective groups are that amateur fights consist of 3 × 3 min rounds (compared to 3 or 5 × 5 min rounds in professional fights), and amateur fighters wear shin guards and a rash guard, and are not permitted to perform certain strikes and holds that are permitted under professional MMA rules. Even though there can be different rule sets in amateur and professional MMA with regards to regulations around the timing of the weigh-in, all of the amateurs in this study competed under rules equivalent to professional MMA rules, i.e., with weigh-in on the day before competition. 

### 2.2. Questionnaire

The questionnaire used in this study was a previously validated Rapid Weight Loss Questionnaire (RWLQ) [20] with slight modifications. The questionnaire has demonstrated good stability, reliability and discriminant validity [20,21], having been conducted with a relatively large and heterogeneous sample—including competitors of both genders—and a wide range of competitive levels and ages. This questionnaire was originally designed for the assessment of RWL in judo athletes, but was then modified and validated for other combat sports [22]. Subsequently, the questionnaire has been modified and utilised for MMA athletes [7,8,14,23,24], and other combat sports [25,26]. Similar to previous work [25], our modifications were to change all instances of “judo” to the combat sport of interest to this study, i.e., “MMA”, and to add questions that better reflected current practices related to MMA such as water loading and hot salt baths [7,16,17,23,24,25,27]. Specifically, we added the option to answer “hot salt baths” and “water loading” under the question “How often did you use each one of the following methods to lose weight before competition?” with the same frequency options of always, sometimes, almost never, never used, and I don’t use anymore. The questionnaire was recreated in Google Forms, and shared as a link to the aforementioned private Facebook page. The questionnaire was open for 8 weeks beginning 1 April 2017, with reminder requests for participation posted to the page once per fortnight.

### 2.3. Data Analysis

The RWLQ was scored as described previously to produce a Rapid Weight Loss Score (RWLS) for each athlete and frequency analysis was performed where appropriate [20]. Our additional questions on water loading and hot salt baths were not scored in the final calculation of RWLS. Therefore, the calculated RWLS remained directly comparable to other studies that employed the RWLQ. One amateur athlete indicated that he had never engaged in RWL and was excluded from the calculation of RWLS. Data were analysed and illustrated using PRISM v7 (GraphPad Software, San Diego, CA, USA). All data were assessed for normality using the Shapiro–Wilk test. For normal distributions, descriptive statistics are reported as mean ± SD, and differences between groups were assessed using an independent samples t-test. For non-normal distributions, descriptive statistics are reported as median (interquartile range) (IQR), and differences between groups were assessed using a Mann–Whitney U test. The significance level was set at α = 0.05 for all tests. Differences between groups are reported as mean (lower 95% confidence interval, upper 95% confidence interval). Effect size was calculated using Cohen’s *d* and interpreted using thresholds of <0.2, ≥0.2, ≥0.5 and ≥0.8 for trivial, small, moderate, and large, respectively.

## 3. Results

Of the n = 30 athletes surveyed, respondents had, on average, 4.7 ± 2.7 y of experience of formally competing in MMA (Table 1), and all but one athlete (97%) had previously engaged in RWL in preparation for competition. The percentage of habitual body mass usually lost in the overall weight cut preparation for a fight averaged 7.9% ± 3.1%, and 100% (85, 133)% of this weight loss was usually regained in the week after a fight (Table 1). In this cohort, the amateur fighters had a lower body mass index (23.6 ± 1.8 vs. 25.0 ± 2.2 kg m^−2^; *p* = 0.030; *d* = 0.70), and tended to have fewer years of competitive experience (3.8 ± 2.6 vs. 5.6 ± 2.6 y; *p* = 0.067; *d* = 0.69).

**Figure 1:**
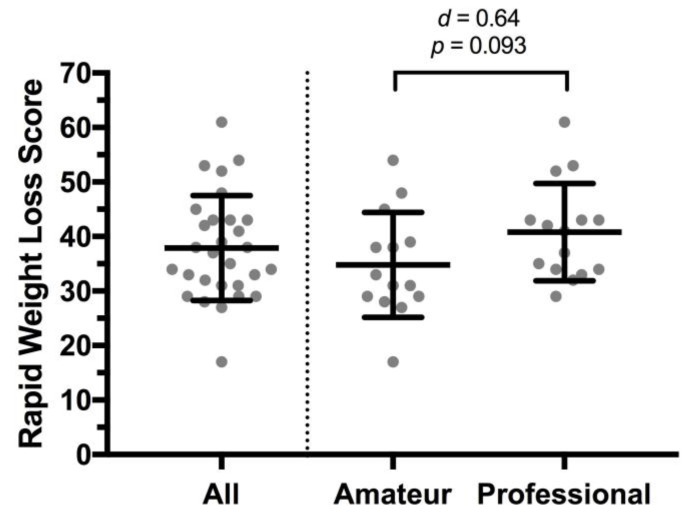
Rapid Weight Loss Score obtained by the RWLQ from the group as a whole (All, n = 29), and based on self-reported status as Amateur (n = 14) or Professional (n = 15). Data bars are mean values with error bars representing standard deviation.

The RWLS for this sample of MMA athletes was 37.9 ± 9.6 (Figure 1). Comparison of RWLS across codes revealed a tendency for higher RWLS [6.0 (−1.1, 13.1); *p* = 0.093] for professional (40.8 ± 8.9) compared to amateur (34.8 ± 9.6), with the magnitude of effect interpreted as ‘moderate’ (*d* = 0.64) (Figure 1).

While energy restriction strategies (i.e., gradual dieting, fasting) are frequently used, methods that reduce body water stores (i.e., water loading, fluid restriction, and hot salt baths) are also commonly employed for RWL by this cohort (Table 2). Water loading was the most commonly used method for RWL, with 90% of the athletes using water loading “sometimes” or “always”. Of those that used water loading, 70% of the athletes start water loading between 5 and 8 days out from the weigh-in. When using water loading, 70% of the athletes consumed between 6 and 9 L of water for the high water intake days. Fluid restriction was used “sometimes” or “always” by 79% of the athletes, with 75% of this number employing the method at between 1 and 24 h prior to weigh-ins. Hot salt baths are commonly used, with 76% of athletes using the method “always” or “sometimes”, compared to 48% of the athletes “always” or “sometimes” using saunas to dehydrate. Gradual dieting was used “sometimes” or “always” by 76% of the athletes, in addition to fasting for 24 h being used “sometimes” or “always” by 55%. Using winter or plastic suits, spitting, laxatives, diuretics, diet pills, and vomiting were the RWL methods that were least commonly used in this cohort.

In the present cohort, athletes receive the majority of their advice about weight-making methods from fellow fighters and their coaches/mentors (Table 3). Fellow fighters were “very influential” to 41% of the athletes, and coaches/mentors were “very influential” to 38% of the athletes in their weight-making practices, with these sources being “somewhat influential” to another 31% and 24% of the athletes, respectively. Very little influence was provided by health and fitness professionals. Doctors, dietitians and physical trainers were said to be “not influential” by 67%, 41%, and 37% of the athletes, respectively.

## 4. Discussion

The present study establishes that a variety of methods for RWL are widely used by MMA athletes at amateur and professional levels. In addition to energy restriction by gradual dieting and short-term fasting, the methods most commonly being employed by this Irish cohort are those that reduce body water stores, i.e., water loading, fluid restriction, and hot salt baths. Even discounting water loading and hot salt baths, RWL scores were higher in these athletes than those reported in other combat sports, and a tendency existed for higher RWL scores in professional compared to amateur fighters. Fellow fighters and coaches are the dominant sources of information on methods of RWL in this cohort of athletes.

Despite the increasing popularity of MMA [12], and the concerns expressed around the safety of weight-making practices in the sport [16,19], there has been a scarcity of studies describing the prevalence and magnitude of RWL by these athletes, or indications of the personnel who are influencing these practices. During the execution of the present study, two other reports emerged describing weight-making practices in MMA in athlete cohorts of n = 70 [23] and n = 314 [24]. The findings of these studies are largely confirmed in our study, but in addition, we report an estimate of prevalence of the use of hot salt baths by MMA athletes.

Hot baths generally describe the practice of hot water immersion (e.g., >38 °C), and supported by “wrapping” in warm towels or bedclothes for a period of time prior to further exposures to hot water immersion [17]. As part of the hot bath protocol, fighters will often add Epsom salts (magnesium sulfate) with the prevailing wisdom that this addition elicits greater loss of body mass through sweating-induced dehydration. Indeed, the addition of a salt to a hot water immersion to produce greater body mass loss does have some empirical evidence to support its practice [28]. Hot baths/hot salt baths have been briefly mentioned as part of weight-making practices in a number of case and small cohort studies [7,9,17,27], but to date, their prevalence in a larger cohort has not been documented. In the present cohort, 76% of the athletes reporting using hot salt baths “always” or “sometimes”, with only one athlete reporting to have “never used” them. Clearly, there is a need for future work to explore the detailed protocols, and outcomes of this method for RWL given this prevalence.

Like other work [23,24], methods that reduce body water stores (i.e., water loading, fluid restriction, and hot salt baths) are the most commonly employed methods for RWL by this cohort. All but one (97%) of the n = 30 of those surveyed lost weight in order to compete, with water loading being the most prevalent method employed at a frequency of “always” or “sometimes” in 90% of respondents. The high prevalence of RWL is consistent with other reports in MMA athletes [23,24], and is greater than that reported, on average, in other combat and weight category sports [22,23,25]. The prevalence of RWL varies considerably between the various combat and weight category sports, with a number of reviews summarising the prevalence as between 50% and 80% [1,3,4]. Combat sports tend to report a higher prevalence of RWL compared to other weight category sports [1,3,4], and the prevalence of RWL in MMA is generally >95% of athletes [23,24]. Similarly, the prevalence of water loading observed in MMA athletes in the present study, and by others [23,24], appears to be higher than the prevalence of water loading reported in other combat sports [23,25]. Differences in methods of RWL between sports are not solely limited to methods to reduce body water stores; for example, the use of fasting “always” or “sometimes” was only reported by 24% of boxers compared to 70% of wrestlers [25]. The specific reasons for differences in methods of RWL between other combat and weight category sports remains to be explored. Several factors are likely to be at play, including the culture of the sport itself, the number of weight categories, and the duration of the time period between weigh-in and competition [1,3,4,5].

The level of competition, calibre of athlete and/or professional status have been observed to varying degrees to be influencing factors in the prevalence and/or magnitude of RWL in several studies [21,23,25], i.e., higher prevalence of RWL, greater %body mass lost, and/or higher RWL scores were associated with more elite performers. A similar tendency was noted in the present study, with a moderate effect size observed for higher RWL score in the professional fighters. The RWL score is an outcome based on scoring of the RWLQ as described by the original validation papers [20,21], which allows for direct comparison between studies. The RWL score for this sample of MMA athletes was 37.9 ± 9.6, which is higher than scores of ~31 reported in boxing, judo, taekwondo, and wrestling [25].

This scoring system and calculated RWL scores do not include a weighting attributed to water loading or hot salt baths, which are common practices by MMA athletes. Whether these methods are commonly used in other combat sports, or whether the prevalence of hot baths reported herein is similar in other MMA cohorts, remains to be confirmed. Nevertheless, separate to the RWL scoring system, it is generally accepted that the %body mass lost as part of the RWL process in greater in MMA than other sports [3,4]. In other combat sports, the %body mass lost during averages ~2% to 6% [22,23,25], whereas the average is ~5% to 10% in MMA [7,8,15,16,23,24]. The 7.9% ± 3.1% reported by our cohort is, therefore, consistent with the magnitudes in the latter studies cited. 

Fellow fighters and coaches/mentors were the most influential sources of information for weight-making practices in this cohort of MMA athletes, whereas health and fitness professionals such as doctors, dietitians and physical trainers are generally reported to have limited influence. This finding is not exclusive to MMA, and in fact, is widely reported across a range of combat and weight category sports [21,23,24,25]. Whether it is possible to overcome ingrained practices in a sport such as MMA remains to be seen, but support staff should be aware of these key influencers of the practices of their athletes. Governing bodies should consider formal education modules for their coaches and athletes on the potential health, safety and performance consequences of methods for RWL.

Aside from the limitations generally associated with self-reported data, another limitation that must be acknowledged in the present study is that the cohort of athletes surveyed were part of the same larger MMA franchise, SBG. Although the athletes trained in several different MMA gyms, the convenience sampling approach using an internal social media page likely resulted in the recruitment of athletes with largely similar coaching and support staff. While circulation of nutrition and weight-making advice is not a feature of the social media page, given the described influence of coaches and fellow fighters on methods of RWL, the sampling approach in this study may have introduced a bias to the results. Specifically, the finding of a high prevalence of hot salt bath use will need to be confirmed in other MMA cohorts. However, the overall results in terms of prevalence, magnitude and methods of RWL are largely similar to that of surveys of larger MMA cohorts [23,24].

Therefore, we conclude that manipulation of body water stores through water loading, fluid restriction and hot salt baths, in addition to gradual dieting and short-term fasting, are the most common methods of RWL employed by MMA athletes. Given the greater degree of RWL in MMA compared to other sports, whether measured by prevalence, %body mass loss or RWL score, there is a need for research on the physiological responses to these methods of RWL in addition to understanding the safety and performance characteristics of athletes who have undertaken aggressive weight regain strategies subsequent to these weight-making practices. Such research will benefit fighters, coaches and administrators alike in developing evidence-based practices, recommendations and policies for the sport. 

## Figures and Tables

**Table 1:** Participant characteristics ^1^.

	All (*n* = 30)	Amateur (*n* = 15)	Professional (*n* = 15)	Amateur vs. Professional *p* Value
**Age** (y)	25.5 ± 4.4	24.3 ± 4.2	26.7 ± 4.4	0.150
**Years of experience competing in MMA** (y)	4.7 ± 2.7	3.8 ± 2.6	5.6 ± 2.6	0.067
**Weight category**	AW, n = 0:	AW, n = 0:	AW, n = 0:	
	SW, n = 0;	SW, n = 0;	SW, n = 0;	
	FLW, n = 2;	FLW, n = 1;	FLW, n = 1;	
	BW, n = 4;	BW, n = 2;	BW, n = 2;	
	FEW, n = 4;	FEW, n = 1;	FEW, n = 3;	
	LW, n = 10;	LW, n = 6;	LW, n = 4;	
	WW, n = 6;	WW, n = 5;	WW, n = 1;	
	MW, n = 3;	MW, n = 0;	MW, n = 3;	
	LHW, n = 0;	LHW, n = 0;	LHW, n = 0;	
	HW, n = 1	HW, n = 0	HW, n = 1	
**Habitual body mass** (kg)	78.3 ± 9.7	76.3 ± 7.3	80.3 ± 11.5	0.257
**Height** (m)	1.79 ± 0.07	1.80 ± 0.07	1.79 ± 0.08	0.743
**Habitual body mass index** (kg m^−2^)	24.3 ± 2.1	23.6 ± 1.8	25.0 ± 2.2	0.030
**Fights in previous 12 months**	2.5 (1.0, 3.3)	2 (1, 4)	1 (1, 3)	0.853
**Usual weight cut** (% of current body mass)	7.9 ± 3.1	7.2 ± 3.4	8.6 ± 2.8	0.397
**Usual weight regain in week after fight** (% of weight cut)	100 (85, 133)	100 (80, 131)	100 (91, 133)	0.612

^1^ Data are reported as mean ± SD or median (IQR) for normal and non-normal distributions, respectively. Weight category abbreviations: AW, atomweight; SW, strawweight; FLW, flyweight; BW, bantamweight; FEW, featherweight; LW, lightweight; WW, welterweight; MW, middleweight; LHW, light heavyweight; HW, heavyweight.

**Table 2:** Frequency analysis of the weight loss methods reported by the mixed martial arts athletes (n = 29).

Method	Always (%)	Sometimes (%)	Almost Never (%)	Never (%)	Do Not Use Anymore (%)
**Gradual dieting**	62.1	24.1	6.9	0.0	6.9
**Skipping one or two meals**	20.7	27.6	24.1	17.2	10.3
**Fasting**	31.0	24.1	10.3	24.1	10.3
**Restricting fluids**	62.1	17.2	10.3	6.9	3.4
**Increased exercise**	34.5	31.0	13.8	17.2	3.4
**Heated training rooms**	13.8	34.5	3.4	48.3	0.0
**Sauna**	27.6	20.7	27.6	10.3	13.8
**Hot salt baths**	34.5	41.4	20.7	3.4	0.0
**Training with rubber/plastic suits**	31.0	13.8	20.7	24.1	10.3
**Using winter or plastic suits**	0.0	3.4	0.0	96.6	0.0
**Spitting**	10.3	17.2	6.9	65.5	0.0
**Laxatives**	3.4	17.2	3.4	72.4	3.4
**Diuretics**	3.4	3.4	0.0	89.7	3.4
**Diet pills**	0.0	3.4	0.0	86.2	10.3
**Vomiting**	0.0	0.0	0.0	100.0	0.0
**Water loading**	62.1	27.6	3.4	6.9	0.0

**Table 3:** Frequency analysis of the individuals who are influential on the weight-making practices reported by the mixed martial arts athletes.

Source	Very Influential (%)	Somewhat Influential (%)	Unsure (%)	A Little Influential (%)	Not Influential (%)
**Online/written Material**	18.5	14.8	14.8	14.8	37.0
**Fellow fighter/training colleague**	41.4	31.0	17.2	3.4	6.9
**Physician/doctor**	3.7	7.4	3.7	18.5	66.7
**Physical trainer**	14.8	11.1	29.6	7.4	37.0
**Coach/mentor**	37.9	24.1	20.7	6.9	10.3
**Parents**	0.0	3.7	3.7	11.1	81.5
**Dietitian**	14.3	14.3	10.7	14.3	46.4
